# 5,6-Diphenyl­pyrazine-2,3-dicarbonitrile

**DOI:** 10.1107/S1600536809033029

**Published:** 2009-08-22

**Authors:** Tuncer Hökelek, Ergin Yalçın, Zeynel Seferoğlu, Ertan Şahin

**Affiliations:** a Hacettepe University, Department of Physics, 06800 Beytepe, Ankara, Turkey; b Gazi University, Department of Chemistry, 06500 Beşevler, Ankara, Turkey; c Atatürk University, Department of Chemistry, 22240 Erzurum, Turkey

## Abstract

In the title compound, C_18_H_10_N_4_, the pyrazine ring is oriented at dihedral angles of 48.08 (7) and 44.80 (7)° with respect to the phenyl rings, while the dihedral angle between the phenyl rings is 49.47 (7)°. In the crystal structure, weak π–π contacts between pyrazine and phenyl rings [centroid–centroid distance = 3.813 (1) Å] may stabilize the structure.

## Related literature

For applications of 2,3-dicyano­pyrazine derivatives, see: Hou *et al.* (1993 ▶); Jaung *et al.* (1996 ▶); Takematsu *et al.* (1981 ▶). For a related structure, see: Zhang *et al.* (2009 ▶). For bond-length data, see: Allen *et al.* (1987 ▶).
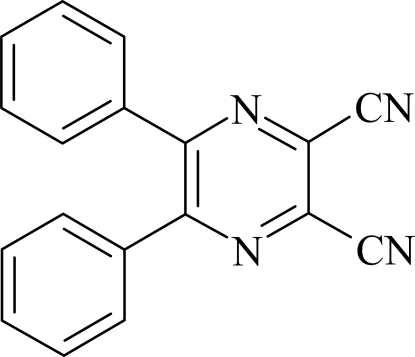


## Experimental

### 

#### Crystal data

C_18_H_10_N_4_
*M* *_r_* = 282.31
Monoclinic, 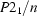

*a* = 9.2195 (2) Å
*b* = 7.2837 (2) Å
*c* = 21.5507 (5) Å
β = 101.108 (1)°
*V* = 1420.06 (6) Å^3^
*Z* = 4
Mo *K*α radiation
μ = 0.08 mm^−1^
*T* = 294 K
0.30 × 0.15 × 0.10 mm

#### Data collection

Rigaku R-AXIS RAPID-S diffractometer
Absorption correction: none
28933 measured reflections
2911 independent reflections
1708 reflections with *I* > 2σ(*I*)
*R*_int_ = 0.137

#### Refinement

*R*[*F* ^2^ > 2σ(*F* ^2^)] = 0.057
*wR*(*F* ^2^) = 0.145
*S* = 1.05
2911 reflections
200 parameters
H-atom parameters constrained
Δρ_max_ = 0.14 e Å^−3^
Δρ_min_ = −0.18 e Å^−3^

### 

Data collection: *CrystalClear* (Rigaku/MSC, 2005 ▶); cell refinement: *CrystalClear*; data reduction: *CrystalClear*; program(s) used to solve structure: *SHELXS97* (Sheldrick, 2008 ▶); program(s) used to refine structure: *SHELXL97* (Sheldrick, 2008 ▶); molecular graphics: *ORTEP-3 for Windows* (Farrugia, 1997 ▶); software used to prepare material for publication: *WinGX* (Farrugia, 1999 ▶) and *PLATON* (Spek, 2009 ▶).

## Supplementary Material

Crystal structure: contains datablocks I, global. DOI: 10.1107/S1600536809033029/xu2592sup1.cif

Structure factors: contains datablocks I. DOI: 10.1107/S1600536809033029/xu2592Isup2.hkl

Additional supplementary materials:  crystallographic information; 3D view; checkCIF report

## Figures and Tables

**Fig. 1.**
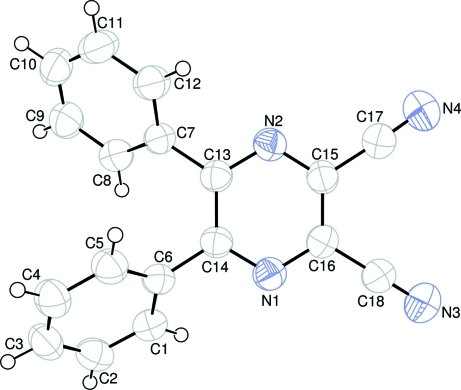
The molecular structure of the title molecule with the atom-numbering scheme. Displacement ellipsoids are drawn at the 50% probability level.

**Fig. 2.**
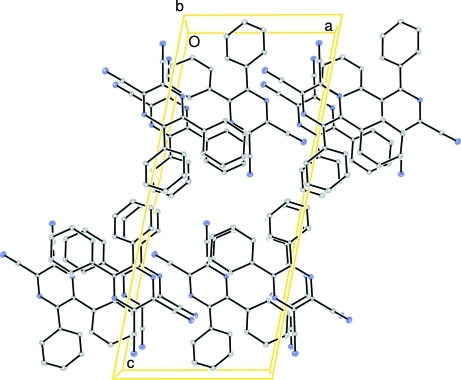
A partial packing diagram.

## supplementary crystallographic information

### Comment

2,3-Dicyanopyrazine derivatives have become a potential subject of investigation because of their wide variety of applications, which include heterocycles for bioactive substances, coloring matters, nonlinear optical (NLO) and electroluminescence (EL) materials (Hou *et al.*, 1993; Jaung *et al.*, 1996). They are also the intermediate compounds to synthesize phthalocyanine dyes, which is nowadays a very important class of dyes. On the other hand, it has been found that a group of 2,3-dicyanopyrazine derivatives have very good herbicidial activity in treatment of the soil of water-submerged paddies, foliage of weeds in the growth period, and the soil of upland farms, these compounds generally tend to form a rigid chemical-treated layer in the surface of the soil, and have the ability to control barnyard grass and other annual and perennial weeds excellently with substantially no phytotoxicity to transplanted rise plants (Takematsu *et al.*, 1981). The present study was undertaken in order to ascertain the crystal structure of the title compound.

In the molecule of the title compound, (Fig. 1), the bond lengths (Allen *et al.*, 1987) and angles are within normal ranges. The cyano groups bond lengths C17—N4 [1.138 (3) Å] and C18—N3 [1.138 (3) Å] are in good agreement with the corresponding values [1.140 (2) and 1.142 (2) Å] reported in 4,5-diaminobenzene-1,2-dicarbonitrile (Zhang *et al.*, 2009). Rings A (C1—C6), B (C7—C12) and C (N1/N2/C13—C16) are, of course, planar and they are oriented at dihedral angles of A/B = 49.47 (7), A/C = 48.08 (7) and B/C = 44.80 (7)°.

In the crystal structure, the π–π contact between the pyrazine and the phenyl rings, *Cg*1—*Cg*2^i^, [symmetry code: (i) 1/2 - *x*, 1/2 + *y*, 1/2 - *z*, where *Cg*1 and *Cg*2 are centroids of the rings C (N1/N2/C13—C16) and A (C1—C6), respectively] may stabilize the structure, with centroid-centroid distance of 3.813 (1) Å.

As can be seen from the packing diagram (Fig. 2), the molecules are stacked along the *b* axis and elongated along the *a* axis.

### Experimental

For the preparation of the title compound, a mixture of benzyl (2.10 g, 10 mmol), diaminomaleonitrile (1.18 g, 11 mmol) and acetic acid (2 ml) in ethanol (20 ml) and water (15 ml) was heated at 348 K overnight. The reaction mixture was cooled, and water (20 ml) was added. The precipitate was filtered and washed with ethanol and then ether. The crude product was dissolved in dichloromethane and treated with activated charcoal. The solid was recrystallized from ethanol to give colorless crystals (yield; 1.97 g, 70%, m.p. 516–518 K).

### Refinement

H atoms were positioned geometrically, with C—H = 0.93 Å, and constrained to ride on their parent atom with *U*_iso_(H) = 1.2*U*_eq_(C).

### Crystal data

**Table:**

C_18_H_10_N_4_	*F*(000) = 584
*M**_r_* = 282.31	*D*_x_ = 1.320 Mg m^−^^3^
Monoclinic, *P*2_1_/*n*	Mo *K*α radiation, λ = 0.71073 Å
Hall symbol: -P 2yn	Cell parameters from 4324 reflections
*a* = 9.2195 (2) Å	θ = 2.3–26.4°
*b* = 7.2837 (2) Å	µ = 0.08 mm^−^^1^
*c* = 21.5507 (5) Å	*T* = 294 K
β = 101.108 (1)°	Block, colorless
*V* = 1420.06 (6) Å^3^	0.30 × 0.15 × 0.10 mm
*Z* = 4	

### Data collection

**Table:**

Rigaku R-AXIS RAPID-S diffractometer	1708 reflections with *I* > 2σ(*I*)
Radiation source: fine-focus sealed tube	*R*_int_ = 0.137
graphite	θ_max_ = 26.4°, θ_min_ = 2.3°
ω scans	*h* = −11→11
28933 measured reflections	*k* = −9→8
2911 independent reflections	*l* = −26→26

### Refinement

**Table:**

Refinement on *F*^2^	Secondary atom site location: difference Fourier map
Least-squares matrix: full	Hydrogen site location: inferred from neighbouring sites
*R*[*F*^2^ > 2σ(*F*^2^)] = 0.057	H-atom parameters constrained
*wR*(*F*^2^) = 0.145	*w* = 1/[σ^2^(*F*_o_^2^) + (0.0434*P*)^2^ + 0.1959*P*] where *P* = (*F*_o_^2^ + 2*F*_c_^2^)/3
*S* = 1.05	(Δ/σ)_max_ < 0.001
2911 reflections	Δρ_max_ = 0.14 e Å^−^^3^
200 parameters	Δρ_min_ = −0.17 e Å^−^^3^
0 restraints	Extinction correction: *SHELXL97* (Sheldrick, 2008), Fc^*^=kFc[1+0.001xFc^2^λ^3^/sin(2θ)]^-1/4^
Primary atom site location: structure-invariant direct methods	Extinction coefficient: 0.039 (5)

### Special details

**Table:**

Experimental. IR (Mattson 1000 F T—IR spectrophotometer, KBr, ν_max_): 3073 cm^-1^ (aromatic C—H), 2238 cm^-1^ (CN), 1515 cm^-1^ (CC). ^1^H-NMR (Bruker-Spectrospin Avance DPX 400 MHz Ultra-Shield): (δ, DMSO-d_6_) 7.40–7.50 p.p.m. (m, 10H, ArH).
Geometry. All e.s.d.'s (except the e.s.d. in the dihedral angle between two l.s. planes) are estimated using the full covariance matrix. The cell e.s.d.'s are taken into account individually in the estimation of e.s.d.'s in distances, angles and torsion angles; correlations between e.s.d.'s in cell parameters are only used when they are defined by crystal symmetry. An approximate (isotropic) treatment of cell e.s.d.'s is used for estimating e.s.d.'s involving l.s. planes.
Refinement. Refinement of *F*^2^ against ALL reflections. The weighted *R*-factor *wR* and goodness of fit *S* are based on *F*^2^, conventional *R*-factors *R* are based on *F*, with *F* set to zero for negative *F*^2^. The threshold expression of *F*^2^ > σ(*F*^2^) is used only for calculating *R*-factors(gt) *etc*. and is not relevant to the choice of reflections for refinement. *R*-factors based on *F*^2^ are statistically about twice as large as those based on *F*, and *R*- factors based on ALL data will be even larger.

### Fractional atomic coordinates and isotropic or equivalent isotropic displacement parameters (Å^2^)

**Table:**

	*x*	*y*	*z*	*U*_iso_*/*U*_eq_	
N1	0.9159 (2)	0.0899 (3)	0.77849 (9)	0.0548 (5)	
N2	1.1240 (2)	−0.0919 (3)	0.72267 (8)	0.0541 (5)	
N3	1.1092 (3)	0.1454 (3)	0.92857 (11)	0.0803 (7)	
N4	1.4193 (3)	−0.1142 (3)	0.84155 (11)	0.0843 (8)	
C1	0.6099 (3)	0.0462 (3)	0.70896 (11)	0.0558 (6)	
H1	0.6212	0.0037	0.7503	0.067*	
C2	0.4703 (3)	0.0730 (3)	0.67328 (13)	0.0642 (7)	
H2	0.3876	0.0446	0.6903	0.077*	
C3	0.4526 (3)	0.1412 (3)	0.61287 (13)	0.0677 (7)	
H3	0.3582	0.1564	0.5888	0.081*	
C4	0.5738 (3)	0.1870 (4)	0.58798 (12)	0.0688 (7)	
H4	0.5615	0.2378	0.5477	0.083*	
C5	0.7134 (3)	0.1579 (3)	0.62258 (11)	0.0617 (7)	
H5	0.7954	0.1887	0.6054	0.074*	
C6	0.7335 (2)	0.0829 (3)	0.68285 (10)	0.0508 (6)	
C7	0.9497 (3)	−0.1528 (3)	0.62762 (10)	0.0517 (6)	
C8	0.8195 (3)	−0.2521 (3)	0.60918 (11)	0.0625 (7)	
H8	0.7527	−0.2615	0.6363	0.075*	
C9	0.7894 (3)	−0.3364 (4)	0.55095 (13)	0.0717 (7)	
H9	0.7027	−0.4035	0.5390	0.086*	
C10	0.8868 (3)	−0.3215 (4)	0.51061 (12)	0.0755 (8)	
H10	0.8650	−0.3765	0.4709	0.091*	
C11	1.0169 (3)	−0.2254 (4)	0.52857 (12)	0.0746 (8)	
H11	1.0829	−0.2163	0.5011	0.090*	
C12	1.0494 (3)	−0.1427 (3)	0.58728 (11)	0.0631 (7)	
H12	1.1382	−0.0803	0.5997	0.076*	
C13	0.9860 (2)	−0.0659 (3)	0.69065 (10)	0.0496 (6)	
C14	0.8827 (2)	0.0355 (3)	0.71825 (10)	0.0490 (5)	
C15	1.1567 (2)	−0.0300 (3)	0.78196 (10)	0.0526 (6)	
C16	1.0523 (2)	0.0551 (3)	0.81061 (10)	0.0521 (6)	
C17	1.3043 (3)	−0.0720 (3)	0.81610 (11)	0.0617 (7)	
C18	1.0848 (3)	0.1071 (3)	0.87641 (12)	0.0592 (6)	

### Atomic displacement parameters (Å^2^)

**Table:**

	*U*^11^	*U*^22^	*U*^33^	*U*^12^	*U*^13^	*U*^23^
N1	0.0558 (12)	0.0556 (12)	0.0531 (12)	0.0020 (9)	0.0111 (9)	−0.0003 (9)
N2	0.0506 (12)	0.0585 (12)	0.0535 (12)	0.0001 (9)	0.0109 (9)	0.0005 (9)
N3	0.0844 (17)	0.0934 (18)	0.0607 (14)	0.0033 (13)	0.0083 (12)	−0.0100 (12)
N4	0.0646 (16)	0.0988 (19)	0.0842 (17)	0.0087 (13)	0.0009 (13)	−0.0086 (13)
C1	0.0601 (15)	0.0541 (14)	0.0556 (14)	0.0004 (12)	0.0168 (11)	−0.0013 (11)
C2	0.0530 (15)	0.0619 (16)	0.0793 (18)	0.0008 (12)	0.0171 (13)	−0.0019 (13)
C3	0.0542 (16)	0.0680 (17)	0.0765 (18)	0.0112 (12)	0.0012 (13)	0.0025 (14)
C4	0.0691 (18)	0.0709 (17)	0.0646 (16)	0.0123 (14)	0.0083 (14)	0.0117 (13)
C5	0.0588 (16)	0.0685 (17)	0.0595 (15)	0.0057 (12)	0.0160 (12)	0.0069 (12)
C6	0.0518 (14)	0.0493 (13)	0.0519 (13)	0.0046 (10)	0.0112 (10)	−0.0019 (10)
C7	0.0533 (14)	0.0545 (14)	0.0472 (13)	0.0067 (11)	0.0096 (11)	−0.0007 (10)
C8	0.0597 (15)	0.0671 (17)	0.0614 (15)	0.0027 (13)	0.0134 (12)	−0.0081 (13)
C9	0.0681 (18)	0.0721 (18)	0.0709 (18)	0.0028 (13)	0.0032 (14)	−0.0142 (14)
C10	0.088 (2)	0.0764 (19)	0.0560 (16)	0.0228 (16)	−0.0024 (15)	−0.0127 (13)
C11	0.087 (2)	0.083 (2)	0.0592 (16)	0.0206 (17)	0.0273 (15)	0.0004 (14)
C12	0.0651 (16)	0.0658 (16)	0.0602 (16)	0.0054 (12)	0.0167 (12)	−0.0008 (12)
C13	0.0503 (13)	0.0514 (14)	0.0480 (13)	−0.0014 (11)	0.0118 (10)	0.0014 (10)
C14	0.0489 (13)	0.0492 (13)	0.0500 (13)	−0.0002 (10)	0.0126 (10)	0.0016 (10)
C15	0.0490 (13)	0.0574 (14)	0.0505 (14)	−0.0016 (11)	0.0072 (10)	0.0011 (11)
C16	0.0556 (15)	0.0526 (14)	0.0476 (13)	−0.0013 (11)	0.0090 (11)	−0.0002 (10)
C17	0.0594 (16)	0.0664 (17)	0.0586 (15)	−0.0014 (13)	0.0095 (13)	−0.0045 (12)
C18	0.0599 (16)	0.0619 (16)	0.0559 (16)	0.0034 (12)	0.0111 (12)	−0.0023 (12)

### Geometric parameters (Å, °)

**Table:**

N1—C14	1.335 (3)	C7—C12	1.383 (3)
N1—C16	1.338 (3)	C8—C9	1.376 (3)
N2—C13	1.339 (3)	C8—H8	0.9300
N2—C15	1.334 (3)	C9—C10	1.369 (4)
C1—C2	1.380 (3)	C9—H9	0.9300
C1—H1	0.9300	C10—C11	1.378 (4)
C2—H2	0.9300	C10—H10	0.9300
C3—C4	1.371 (4)	C11—H11	0.9300
C3—C2	1.374 (3)	C12—C11	1.381 (3)
C3—H3	0.9300	C12—H12	0.9300
C4—H4	0.9300	C13—C7	1.477 (3)
C5—C4	1.374 (3)	C14—C13	1.422 (3)
C5—H5	0.9300	C15—C16	1.386 (3)
C6—C1	1.391 (3)	C15—C17	1.450 (3)
C6—C5	1.388 (3)	C17—N4	1.138 (3)
C6—C14	1.480 (3)	C18—N3	1.138 (3)
C7—C8	1.393 (3)	C18—C16	1.442 (3)
			
C14—N1—C16	117.58 (19)	C8—C9—H9	119.9
C15—N2—C13	117.57 (19)	C10—C9—C8	120.1 (3)
C2—C1—C6	119.8 (2)	C10—C9—H9	119.9
C2—C1—H1	120.1	C9—C10—C11	120.2 (2)
C6—C1—H1	120.1	C9—C10—H10	119.9
C1—C2—H2	119.8	C11—C10—H10	119.9
C3—C2—C1	120.5 (2)	C10—C11—C12	120.1 (3)
C3—C2—H2	119.8	C10—C11—H11	119.9
C2—C3—H3	119.9	C12—C11—H11	119.9
C4—C3—C2	120.1 (2)	C7—C12—H12	120.0
C4—C3—H3	119.9	C11—C12—C7	120.0 (3)
C3—C4—C5	119.9 (2)	C11—C12—H12	120.0
C3—C4—H4	120.0	N2—C13—C7	115.93 (19)
C5—C4—H4	120.0	N2—C13—C14	120.28 (19)
C4—C5—C6	120.7 (2)	C14—C13—C7	123.8 (2)
C4—C5—H5	119.6	N1—C14—C6	116.61 (19)
C6—C5—H5	119.6	N1—C14—C13	120.96 (19)
C1—C6—C14	120.0 (2)	C13—C14—C6	122.43 (19)
C5—C6—C1	118.8 (2)	N2—C15—C16	122.0 (2)
C5—C6—C14	121.2 (2)	N2—C15—C17	115.6 (2)
C8—C7—C13	121.0 (2)	C16—C15—C17	122.2 (2)
C12—C7—C8	119.2 (2)	N1—C16—C15	121.2 (2)
C12—C7—C13	119.7 (2)	N1—C16—C18	117.1 (2)
C7—C8—H8	119.9	C15—C16—C18	121.7 (2)
C9—C8—C7	120.2 (2)	N4—C17—C15	176.3 (3)
C9—C8—H8	119.9	N3—C18—C16	178.8 (3)
			
C16—N1—C14—C6	−177.30 (19)	C12—C7—C8—C9	−1.2 (4)
C16—N1—C14—C13	3.9 (3)	C13—C7—C8—C9	−178.2 (2)
C14—N1—C16—C15	1.6 (3)	C8—C7—C12—C11	2.2 (4)
C14—N1—C16—C18	−177.3 (2)	C13—C7—C12—C11	179.3 (2)
C15—N2—C13—C14	4.2 (3)	C7—C8—C9—C10	−0.6 (4)
C15—N2—C13—C7	−174.0 (2)	C8—C9—C10—C11	1.4 (4)
C13—N2—C15—C16	1.3 (3)	C9—C10—C11—C12	−0.3 (4)
C13—N2—C15—C17	176.1 (2)	C7—C12—C11—C10	−1.4 (4)
C6—C1—C2—C3	−2.1 (4)	N2—C13—C7—C12	−43.6 (3)
C4—C3—C2—C1	−1.4 (4)	N2—C13—C7—C8	133.4 (2)
C2—C3—C4—C5	2.6 (4)	C14—C13—C7—C8	−44.7 (3)
C6—C5—C4—C3	−0.2 (4)	C14—C13—C7—C12	138.3 (2)
C5—C6—C1—C2	4.3 (3)	N1—C14—C13—N2	−7.0 (3)
C14—C6—C1—C2	−173.3 (2)	N1—C14—C13—C7	171.0 (2)
C1—C6—C5—C4	−3.2 (4)	C6—C14—C13—N2	174.2 (2)
C14—C6—C5—C4	174.5 (2)	C6—C14—C13—C7	−7.8 (3)
C1—C6—C14—N1	−49.0 (3)	N2—C15—C16—N1	−4.4 (4)
C1—C6—C14—C13	129.8 (2)	N2—C15—C16—C18	174.4 (2)
C5—C6—C14—N1	133.4 (2)	C17—C15—C16—N1	−178.9 (2)
C5—C6—C14—C13	−47.8 (3)	C17—C15—C16—C18	0.0 (4)
